# Transplantation of tauroursodeoxycholic acid–inducing M2‐phenotype macrophages promotes an anti‐neuroinflammatory effect and functional recovery after spinal cord injury in rats

**DOI:** 10.1111/cpr.13050

**Published:** 2021-05-07

**Authors:** Gong Ho Han, Seong Jun Kim, Wan‐Kyu Ko, Daye Lee, In‐Bo Han, Seung Hun Sheen, Je Beom Hong, Seil Sohn

**Affiliations:** ^1^ Department of Neurosurgery CHA Bundang Medical Center CHA University Seongnam‐si Korea; ^2^ Department of Biomedical Science CHA University Seongnam‐si Korea; ^3^ Department of Neurosurgery Kangbuk Samsung Hospital Sungkyunkwan University School of Medicine Seoul Korea

**Keywords:** bone marrow–derived macrophages, neuroinflammation, spinal cord injuries, tauroursodeoxycholic acid, transplantation

## Abstract

**Objectives:**

In this study, we study the transplantation of tauroursodeoxycholic acid (TUDCA)‐induced M2‐phenotype (M2) macrophages and their ability to promote anti‐neuroinflammatory effects and functional recovery in a spinal cord injury (SCI) model.

**Methods:**

To this end, compared to the granulocyte‐macrophage colony‐stimulating factor (GM‐CSF), we evaluated whether TUDCA effectively differentiates bone marrow–derived macrophages (BMDMs) into M2 macrophages.

**Results:**

The M2 expression markers in the TUDCA‐treated BMDM group were increased more than those in the GM‐CSF‐treated BMDM group. After the SCI and transplantation steps, pro‐inflammatory cytokine levels and the mitogen‐activated protein kinase (MAPK) pathway were significantly decreased in the TUDCA‐induced M2 group more than they were in the GM‐CSF‐induced M1 group and in the TUDCA group. Moreover, the TUDCA‐induced M2 group showed significantly enhanced tissue volumes and improved motor functions compared to the GM‐CSF‐induced M1 group and the TUDCA group. In addition, biotinylated dextran amine (BDA)–labelled corticospinal tract (CST) axons and neuronal nuclei marker (NeuN) levels were increased in the TUDCA‐induced M2 group more than those in the GM‐CSF‐induced M1 group and the TUDCA group.

**Conclusions:**

This study demonstrates that the transplantation of TUDCA‐induced M2 macrophages promotes an anti‐neuroinflammatory effect and motor function recovery in SCI. Therefore, we suggest that the transplantation of TUDCA‐induced M2 macrophages represents a possible alternative cell therapy for SCI.

## Graphical abstract

Bone marrow–derived monocytes were differentiated into M2‐phenotype (M2) macrophages using tauroursodeoxycholic acid (TUDCA). TUDCA‐induced M2 macrophages were directly transplanted to a lesion in an injured spinal cord. The pro‐inflammatory cytokine levels were significantly decreased in the TUDCA‐induced M2 group. We suggest that the transplantation of TUDCA‐induced M2 macrophages represents a possible alternative cell therapy for SCI.

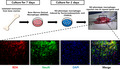

## 1 INTRODUCTION

Spinal cord injuries (SCIs) occur as a result of fractures, dislocations and compressed vertebra. The annual incidence of SCI in the United States alone is estimated to be 12,000 patients.^1^ SCI patients suffer from dysfunctions of the central nervous system (CNS) and the peripheral nervous system (PNS).^2^

Methylprednisolone (MP) has been used for the treatment of SCI patients.^3^ However, MP has side effects such as wound infections, pneumonia and myopathy.^4^ Therefore, the use of MP as a SCI treatment remains controversial.^5^ Tauroursodeoxycholic acid (TUDCA) is a natural molecule containing taurine conjugated with ursodeoxycholic acid. It has been in use since ancient times as a component of traditional Asian medicine.^6^ TUDCA is a hydrophilic nontoxic bile acid that is produced endogenously at low levels in humans and bears.^7^ Food and Drug Administration (FDA) approved TUDCA for the treatment of liver diseases such as cirrhosis and hepatitis.^8^ Moreover, TUDCA has protective effects in those with CNS diseases such as Huntington's disease and Alzheimer's disease.^9^, ^10^, ^11^ In previous studies by the authors, we suggested that TUDCA can be an alternative drug based on its anti‐neuroinflammatory effects in macrophages and in SCI rats.^6^, ^12^

Secondary injury reactions occur in SCI as a result of activated macrophages.^13^ During the inflammatory reaction, M1‐phenotype (M1) macrophages release inflammatory cytokines, including tumour necrosis factor‐α (TNF‐α), interleukin (IL)‐1β and IL‐6.^14^, ^15^ On the other hand, M2‐phenotype (M2) macrophages express macrophage mannose receptors (cluster of differentiation 206, CD206) and arginase‐1 (Arg‐1) and secrete anti‐inflammatory cytokine (IL‐4).^13^, ^16^ Therefore, M2 macrophages facilitate cellular processes such as tissue repair.^17^

Hence, we hypothesized that TUDCA‐induced M2 macrophages can promote spinal cord repair because they limit secondary injuries.

## 2 MATERIALS AND METHODS

### 2.1 Materials

The TUDCA used here was obtained from TCI (Tokyo Chemical Industry Co.). It was solubilized in Dulbecco's modified Eagle's medium (DMEM, GIBCO) containing 10% foetal bovine serum (FBS, GIBCO) and 1% penicillin‐streptomycin (PS, GIBCO). Dulbecco's phosphate‐buffered saline (DPBS) was purchased from GIBCO (Life Technologies).

### 2.2 Isolation and primary culture of bone marrow–derived macrophages (BMDMs)

**FIGURE 1:**
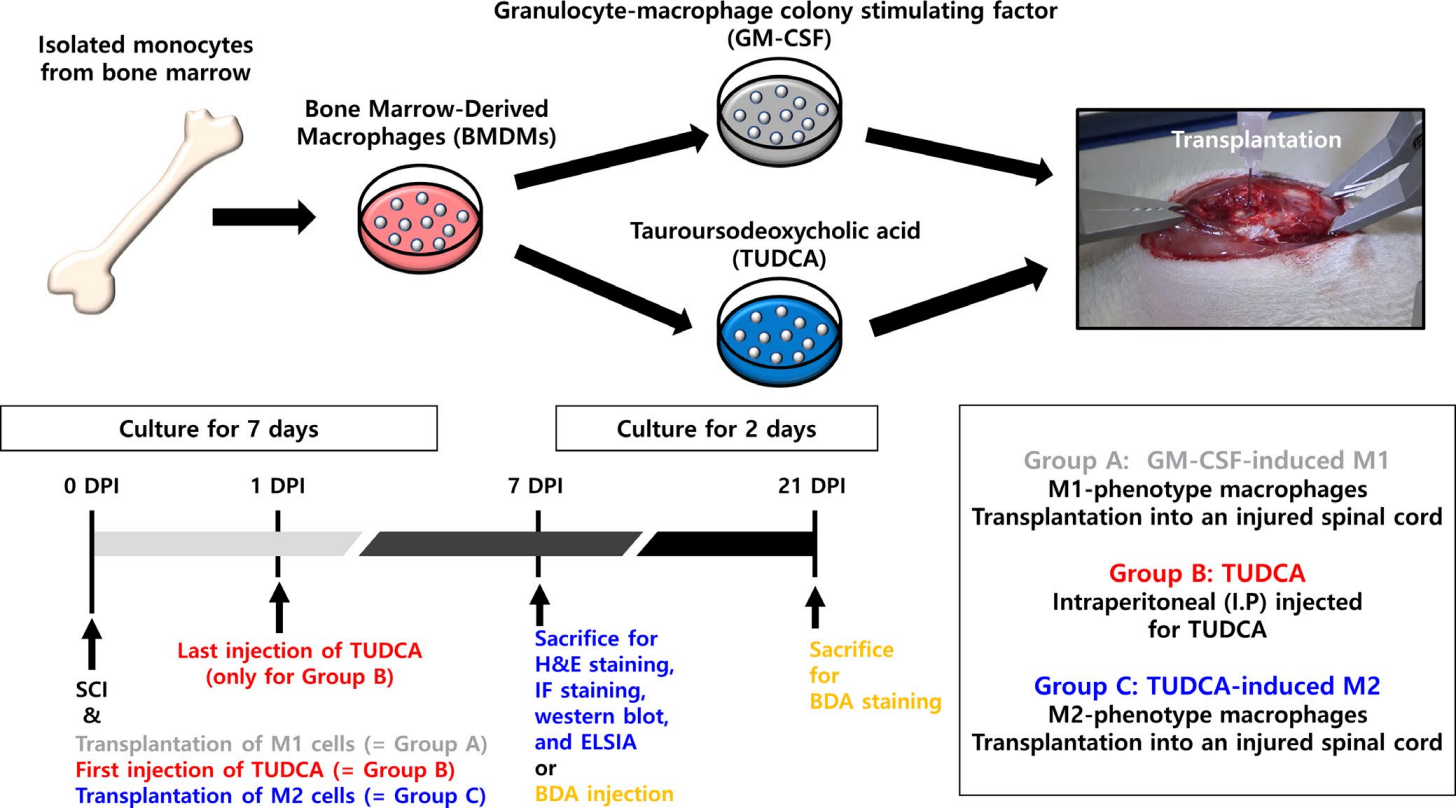
Schematic procedures of the transplantation therapy of tauroursodeoxycholic acid (TUDCA)–induced M2‐phenotype macrophages in the contusion spinal cord injury (SCI) rat model in this study

To obtain rat BMDMs, bone marrow (BM) cells derived from femurs and tibias were harvested and cultured.^18^ The bone inner cavity was rinsed with 10 mL of a medium containing DMEM, 10% FBS, 2% glutamate and 1% PS to harvest BM cells. Collected BM cells were centrifuged (10 minutes, 450 × *g*). Erythrocytes were lysed in a red blood lysis buffer (Cat. 37757; Sigma). To exclude resident macrophages, cells were incubated for 4 hours at 37℃ in culture‐treated petri dish (Cat. 430591; Corning). Afterwards, the supernatant was collected and centrifugated (10 minutes, 450 × *g*). The pellet was dissociated in 150 mL complete DMEM (cDMEM) containing 10% FBS, 2% glutamate, 1% PS and L929‐conditioned medium.^19^ 10 mL of suspended cells was distributed to a petri dish. The dish was cultivated at 37℃ in 5% CO_2_. After 3 days, we added 10 mL of cDMEM to each petri dish and the cells were incubated for another 4 days. Finally, the BMDMs were harvested and seeded for the following experiments (Figure 1).

### 2.3 Immunocytochemistry (ICC) staining

We conducted ICC staining to quantify differentiated M1 and M2 macrophages. Macrophages were cultured for 48 hours in DMEM with granulocyte‐macrophage colony‐stimulating factor (GM‐CSF) or TUDCA. The culture medium was removed, and subsequently, cells were fixed with 4% paraformaldehyde (PFA). Fixed cells were treated with anti‐CD86 antibody (1:200; Cat. ab53004; Abcam) and the anti‐mannose receptor antibody (CD206; 1:200; Cat. ab8918; Abcam) at 4℃ overnight. Afterwards, Alexa 488–conjugated (1:200; Cat. A21202; Invitrogen) or Alexa 647–conjugated (1:200; Cat. A21447; Invitrogen) secondary antibody was stained for 1 hour at room temperature. After washing remaining secondary antibodies, the nuclei were stained with 4′,6′‐diamidino‐2‐phenylindole dihydrochloride (DAPI). The fluorescent intensity of ICC staining was detected using a Zeiss LSM 880 confocal microscope.

### 2.4 Fluorescence‐activated cell sorting (FACS) analysis

A FACS analysis of the GM‐CSF or TUDCA‐treated BMDMs was used for macrophage differentiation. The cells were detached with ice‐cold phosphate‐buffered saline (PBS) containing 0.02% ethylenediaminetetraacetic acid (EDTA). 1 × 10^6^ BMDMs were incubated with primary antibodies, including anti‐liver Arg‐1 antibodies (Cat. Ab92274; Abcam), anti‐CD68 antibody (Cat. Ab955; Abcam) and anti‐CD206 antibody (Cat. Ab8918; Abcam) for 1 hour. After one washing step, BMDMs per 1 mL of stain buffer were stained with 5 μL Alexa 488 (Cat. A21202; Invitrogen) and Alexa 647 (Cat. A21447; Invitrogen). The marker expression was analysed on a CytoFLEX V5‐B5‐R3 Flow Cytometer (Beckman Coulter), and data were analysed using the CytExpert software (Beckman Coulter).^20^, ^21^

### 2.5 Development of a SCI animal model

Thirty female Sprague‐Dawley rats (210‐250 g) were divided into the GM‐CSF‐induced M1 group (n = 10), the TUDCA group (n = 10) and TUDCA‐induced M2 group (n = 10). All experimental procedures of handling rats were operated under regulations of the Institutional Animal Care and Use Committee of CHA University (IACUC190162) and according to the Guide for the Care and Use of Laboratory Animals (National Institutes of Health (NIH)).

Fifty mg/kg of Zoletil (Virbac Laboratories) and 10 mg/kg Rompun (Bayer Animal Health Co) were intraperitoneally injected for anesthetizing rats. A midline incision was made on the back. To expose the dura thoracic 9, total laminectomy was performed. A 40 g rod (2.5 mm in diameter) from 30.5 mm height was dropped on the dorsal surface of the spinal cord. Right after the injury, GM‐CSF‐induced M1 and TUDCA‐induced M2 groups received engraftment of 5 μL cell suspension with 1 × 10^6^ GM‐CSF‐induced M1 and TUDCA‐induced M2 macrophages, respectively, into the epicentre of the injured spinal cord using a 26‐gauge microlitre syringe (Cat. 7639‐01; Hamilton). For the TUDCA group, 200 mg/kg of TUDCA was injected immediately after the injury. This was repeated 24 hours after the injury (Figure 1).

All rats were housed in a pathogen‐free facility with controlled temperature and humidity. Rats were allowed free access to food. On each day at 8 am and 8 pm, urination was manually performed. All surgeries were operated by the same spine neurosurgeon (S. Sohn).

### 2.6 Enzyme‐linked immunosorbent assay (ELISA)

Ten mm epicentre segment of the spinal cord lesion was obtained 7 days after the SCI. The collected segments were then homogenized in a 1X RIPA buffer. The segments were then centrifugated at 15 000 RPM for 10 minutes at 4℃. The protein concentration was calculated using a BCA protein analysis kit (Thermo Scientific).^22^ Protein levels were measured using ELISA kits (Koma Biotech).

### 2.7 Western blotting

Equal amounts of protein (30 μg) were separated via sodium dodecyl sulphate‐polyacrylamide gel electrophoresis (SDS‐PAGE) and transferred to nitrocellulose membranes. The membranes were incubated in 5% bovine serum albumin for 1 hour to avoid non‐specific binding. They were probed with primary antibodies with phosphorylated forms of extracellular signal‐regulated kinase (ERK) (p‐ERK; 1:1000; Cat. 4377S), c‐Jun N‐terminal kinase (JNK) (p‐JNK; 1:1000; Cat. 4668S) and p38 (p‐p38; 1:1000; Cat. 9211S). Subsequently, equal membranes were stripped and reprobed with the total forms of ERK (t‐ERK; 1:1000; Cat. 9102S), JNK (t‐JNK; 1:100; Cat. 9258S) and p38 (t‐p38; 1:1000; Cat. 9212S). All primary antibodies were purchased from Cell Signaling Technology except for β‐actin (1:5000; ABM). As an internal control, β‐actin was also probed into the membranes. All of the primary antibodies were then incubated with secondary antibodies (1:5000, Gene Tex). The visualized signal bands were detected using an ECL solution (Amersham) through a G: Box Chemi‐XX6 gel doc system (Syngene). The p/t form volumes for the predetermined days were calculated and quantified using ImageJ software (NIH).

### 2.8 Haematoxylin & eosin (H&E) staining

At 7 days after SCI, the rats were anesthetized. Rapid perfusion was performed using ice‐cold saline after cannulation of the left ventricular‐ascending aorta. When the efflux became clear, 4% PFA/PBS was perfused for 5 minutes.^22^ The 10 mm spinal cord segments of the lesion epicentres were collected and fixed overnight in 4% PFA/PBS. They were dehydrated, and paraffin embedding was followed.^23^ Sagittal sections were cut to a thickness of 5 μm. Sections were stained using haematoxylin and eosin. The changes in morphology were observed under a light microscope (IX71: Olympus).

### 2.9 Behavioural tests

Basso, Beattie and Bresnahan **(**BBB) scores for hindlimb function were measured using open‐field locomotion.^24^ The rats were evaluated on 1, 3, 5, 7, 9, 12, 14, 16, 18 and 21 via BBB tests. Two trained investigators who were blind to the experimental conditions performed the behavioural analyses.

### 2.10 Immunofluorescence staining

According to standard procedures for immunofluorescence staining, sections were incubated in a blocking solution to prevent any non‐specific binding reaction for 1 hour. Afterwards, primary antibodies were treated at 4°C overnight. The antibodies used were the anti‐CD206 (1:200; Cat. ab8918; Abcam) and polyclonal anti‐rabbit glial fibrillary acidic protein (GFAP) (1:200; Cat. ab16997; Abcam). The slides were then stained with fluorescent secondary Alexa 488 (Cat. R37114; Invitrogen) and Alexa 568 (Cat. A10042; Invitrogen) in a blocking solution (1:200; Invitrogen) for 2 hours at room temperature. Afterwards, the nuclei were stained with DAPI. Sections were washed in PBS and then mounted with a specific medium (DakoCytomation). Confocal images were acquired using a Zeiss LSM 880 confocal microscope. Quantification of the fluorescent intensity was carried out by ImageJ software.

### 2.11 Corticospinal tract (CST) tracing by biotinylated dextran amine (BDA)

Tract tracing of axons was performed via BDA injections (Cat. D1956; Invitrogen). The BDA was injected into two sites (one on each side of the cord, 0.5 μL (dissolved in sterile saline)) 1.0 mm below the surface at 0.1 μL per minute using a 33‐gauge Hamilton syringe (Cat. 7635‐01; Hamilton). At 2 weeks after the BDA injection, they were stained with primary antibodies at 4°C overnight. The antibodies used here were the recombinant anti‐NeuN antibody (1:200; Cat. ab177487; Abcam). The slides were then incubated with fluorescent secondary Alexa 488 (Cat. A11034; Invitrogen) and Alexa 594 (Cat. S32356; Invitrogen) in a blocking solution (1:200; Invitrogen) for 2 hours at room temperature. Afterwards, the nuclei were stained with DAPI. Sections were washed in PBS and then mounted with a specific medium (DakoCytomation). Confocal images were acquired using a Zeiss LSM 880 confocal microscope. Quantification of the fluorescent intensity was carried out using ImageJ software.

### 2.12 Statistical analyses

All values were presented as the mean ± standard deviation (SD). A one‐way analysis of variance (ANOVA) followed by a post hoc test was used to verify statistical differences among the groups. Behavioural scores were analysed by Student's *t*‐tests. Differences with *P*‐values for which *P < .05, **P < .01 and ***P < .001 were considered as statistically significant.

## 3 RESULTS

### 3.1 TUDCA treatment increases the number of M2 macrophages on BMDMs

**FIGURE 2:**
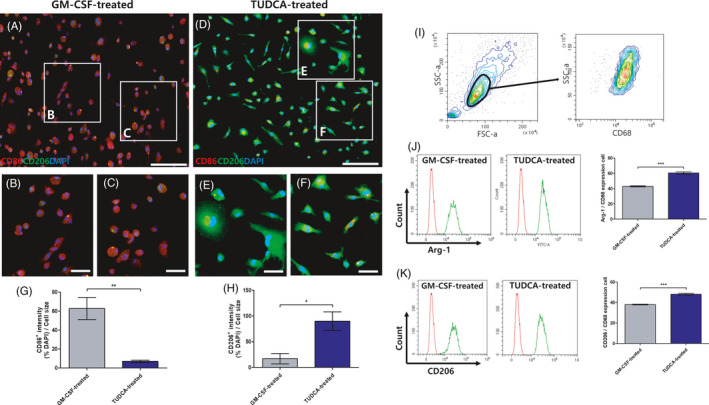
Immunocytochemistry staining and fluorescence‐activated cell sorting of bone marrow–derived macrophages (BMDMs) induced with GM‐CSF and TUDCA. (A‐C) Granulocyte‐macrophage colony‐stimulating factor (GM‐CSF)–treated BMDM immunocytochemistry staining of CD86 and CD206, and (D‐F) TUDCA‐treated BMDM immunocytochemistry staining of CD86 and CD206. Scale bar, 50 μm (A, D) and 10 μm (B‐C), (E‐F). (G) Quantitative analysis of the CD86 fluorescence intensity levels. (H) Quantitative analysis of the CD206 fluorescence intensity levels. (I) Images showing representative forward scatter (FSC) and side scatter (SSC) plots and the gate used for the subsequent analysis, (J) arginase‐1 (Arg‐1)‐positive (green) BMDM population selected for analysis among CD68‐positive (red) BMDMs. (K) CD206‐positive (green) BMDM population selected for analysis among CD68‐positive (red) BMDMs. Results are the mean ± error of the mean (SEM) of triplicate experiments: ^*^P < .05, **P < .01 and ^***^P < .001

To observe whether TUDCA upregulates M2 differentiation, we stained BMDMs in the GM‐CSF‐treated group and the TUDCA‐treated group by ICC staining (Figure 2A‐H). M1 and M2 macrophages are commonly associated with the expression of surface antigens such as CD86 and CD206, respectively.^25^ After 2 days, the CD86 expression levels in the TUDCA‐treated group were lower than those in the GM‐CSF‐treated group (Figure 2G; GM‐CSF‐treated group vs TUDCA‐treated group: 62.81 ± 20.23 vs 6.97 ± 2.06; ^**^P < .01). Furthermore, the CD206 expression levels in the TUDCA‐treated group were higher than those in the GM‐CSF‐treated group (Figure 2H; GM‐CSF‐treated group vs TUDCA‐treated group: 16.94 ± 17.25 vs 89.91 ± 31.49; ^*^P < .05).

To determine whether TUDCA can express M2 macrophages from BMDMs, we conducted FACS after 2 days (Figure 2I‐K). M2 macrophages can express CD206 and Arg‐1.^26^ The Arg‐1 expression levels in the TUDCA‐treated group were significantly increased compared to those in the GM‐CSF‐treated group (Figure 2J; GM‐CSF‐treated group vs TUDCA‐treated group: 42.96 ± 1.61 vs 60.41 ± 2.80; ^***^P < .001). Moreover, the CD206 expression levels in the TUDCA‐treated group were significantly increased relative to those in the GM‐CSF‐treated group (Figure 2K; GM‐CSF‐treated group vs TUDCA‐treated group: 38.08 ± 0.73 vs 48.08 ± 1.55; ^***^P < .001).

### 3.2 Transplantation of TUDCA‐induced M2 macrophages inhibits inflammatory cytokines and increases anti‐inflammatory cytokine

**FIGURE 3:**
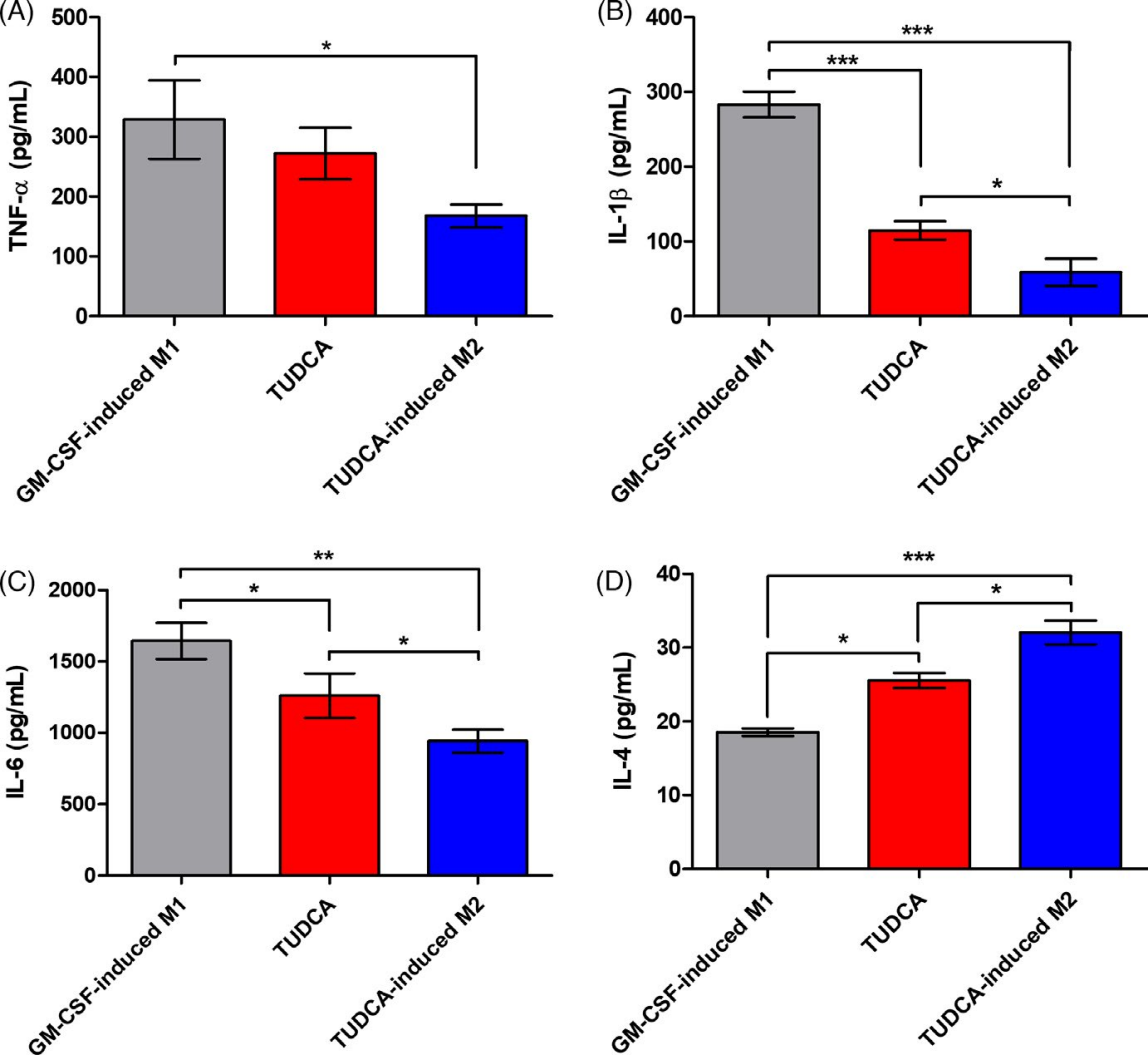
Enzyme‐linked immunosorbent assays (ELISAs). The protein secretion levels of the injured spinal cord segments are shown in the GM‐CSF‐induced M1, TUDCA and TUDCA‐induced M2 groups at 7 days. The relative secretion levels of (A) tumour necrosis factor‐α (TNF‐α), (B) interleukin (IL)‐1β, (C) IL‐6 and (D) IL‐4 were measured according to the directions provided by the manufacturer of each kit. Results are the mean ± SEM of triplicate experiments ^*^P < .05, ^**^P < .01 and ^***^P < .001

For further verification of the anti‐neuroinflammatory effect of the TUDCA‐induced M2 macrophages, we measured the inflammatory cytokines and anti‐inflammatory cytokine by ELISA (Figure 3A‐D). Seven days after SCI, the TNF‐α, IL‐1β and IL‐6 secretion levels in the TUDCA‐induced M2 group were significantly inhibited relative to those in the GM‐CSF‐induced M1 group (Figure 3A‐C; ^*^P < .05, ^**^P < .01 and ^***^P < .001). It was also found that the IL‐1β and IL‐6 secretion levels in the TUDCA‐induced M2 group were significantly inhibited compared to those in the TUDCA group (Figure 3B,C; ^*^P < .05). On the other hand, the IL‐4 secretion level in the TUDCA‐induced M2 group was significantly increased compared to those in the GM‐CSF‐induced M1 group and the TUDCA group (Figure 3D; **P < .01 and ***P < .001).

### 3.3 Transplantation of TUDCA‐induced M2 macrophages inhibits the phosphorylation of ERK, JNK and p38 in the mitogen‐activated protein kinase (MAPK) pathway

**FIGURE 4:**
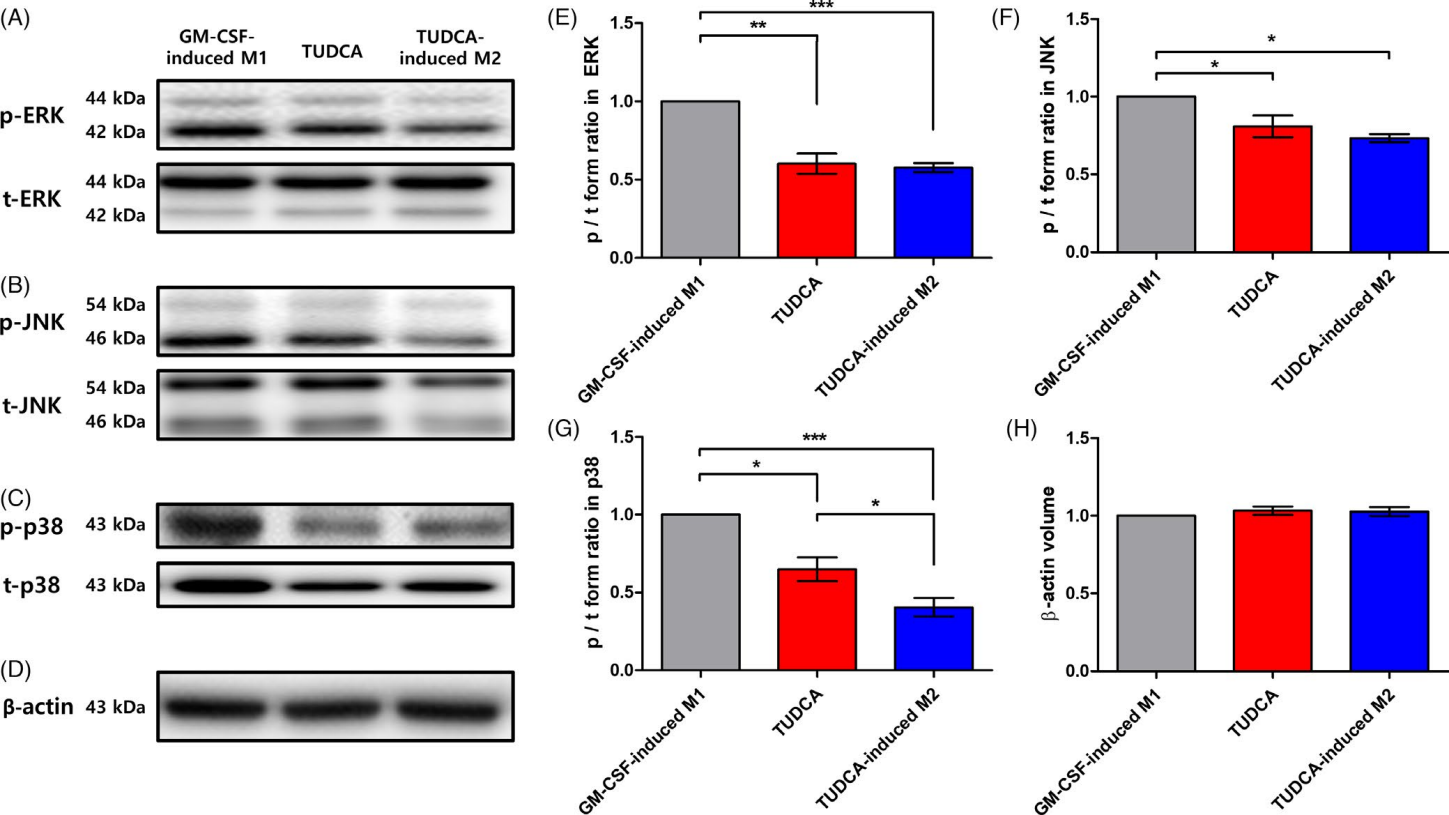
Western blot. Transplantation effects of TUDCA‐induced M2 macrophages on the phosphorylation of extracellular signal‐regulated kinase (ERK), c‐Jun N‐terminal kinase (JNK) and p38. Injured spinal cord segments were collected for the GM‐CSF‐induced M1, TUDCA and TUDCA‐induced M2 groups at seven days after SCI. The p/t form volume in the GM‐CSF‐induced M1 group was set to 1‐fold, and the ratio was relatively calculated and quantified. Images of the p and t forms of (A) ERK, (B) JNK, (C) p38 and (D) β‐actin. Quantitative analyses of the p/t forms of (E) ERK, (F) JNK, (G) p38 and (H) β‐actin. Results are the mean ± SEM of triplicate experiments: ^*^P < .05, ^**^P < .01 and ^***^P < .001

The phosphorylation activities of ERK, JNK and the p38 signals in the MAPK pathway are key processes during the inflammatory response after SCI (Figure 4A‐G). As shown in Figure 4A, the phosphorylated forms per total form (p/t) volume of ERK in the TUDCA‐induced M2 group was significantly decreased relative to that in the GM‐CSF‐induced M1 group (Figure 4E; GM‐CSF‐induced M1 group vs TUDCA‐induced M2 group: 1.00 vs 0.58 ± 0.05; ^***^P < .001). The p/t volume of JNK in the TUDCA‐induced M2 group was also significantly decreased compared to that in the GM‐CSF‐induced M1 group (Figure 4F; GM‐CSF‐induced M1 group vs TUDCA‐induced M2 group: 1.00 vs 0.73 ± 0.04; ^*^P < .05). The p/t volume of p38 in the TUDCA‐induced M2 group was significantly decreased compared to that in the GM‐CSF‐induced M1 group and TUDCA group (Figure 4G; GM‐CSF‐induced M1 group: 1.00, TUDCA group: 0.65 ± 0.13 and TUDCA‐induced M2 group: 0.40 ± 0.10; ^*^P < .05 and ^***^P < .001). The p/t volumes of β‐actin, an internal control, were 1.00 (GM‐CSF‐induced M1 group), 1.03 ± 0.05 (TUDCA group) and 1.02 ± 0.05 (TUDCA‐induced M2 group) (Figure 4H).

### 3.4 Transplantation of TUDCA‐induced M2 macrophages enhances tissue repair and improves motor function after SCI

**FIGURE 5:**
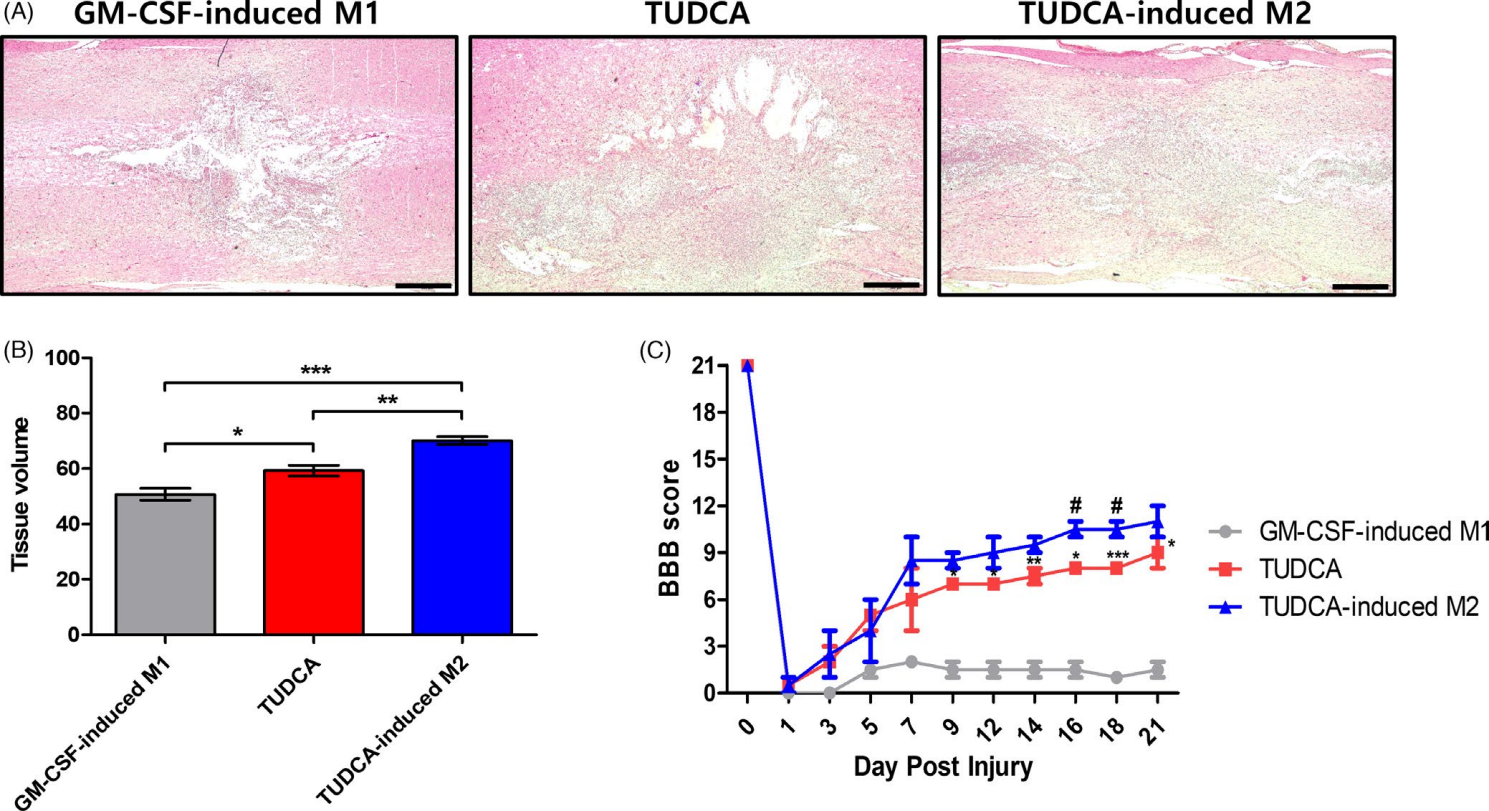
Haematoxylin and eosin staining and motor function recovery. A, Seven days after SCI, the histological structures of the tissue volume are shown in GM‐CSF‐induced M1, TUDCA and TUDCA‐induced M2 groups (scale bar = 500 μm). B, Quantitative analysis of the tissue volume. The tissue volume of the mean ± SEM of triplicate experiments: ^*^P < .05, ^**^P < .01 and ^***^P < .001. C, Basso, Beattie and Bresnahan (BBB) locomotor scores were evaluated in the GM‐CSF‐induced M1, TUDCA and TUDCA‐induced M2 groups for 21 d after SCI. The results for the TUDCA group show a significant difference as compared to those for the GM‐CSF‐induced M1 group. Results are the mean ± standard deviation (SD): ^*^P < .05, ^**^P < .01 and ^***^P < .001. The results of TUDCA‐induced M2 group show a significant difference as compared to those of the TUDCA group (*^#^P* < .05)

To observe whether TUDCA‐induced M2 transplantation upregulates the tissue repair effect after SCI, we stained spinal cord tissues in the sham, simple injury, GM‐CSF‐induced M1, TUDCA and TUDCA‐induced M2 groups by H&E staining (Figure 5A,B and Figure S1). Seven days after SCI, the tissue volume in the TUDCA‐induced M2 group was enhanced compared to those in the GM‐CSF‐induced M1 group and the TUDCA group (Figure 5B; GM‐CSF‐induced M1 group: 66.65 ± 2.60, TUDCA group: 66.65 ± 2.60 and TUDCA‐induced M2 group: 66.65 ± 2.60; ^*^P < .05, ^**^P < .01, and ^***^P < .001). The tissue volume in the sham and simple injury groups was detected at 90.86 ± 1.55 and 39.64 ± 3.49, respectively (Figure S1).

**FIGURE 6:**
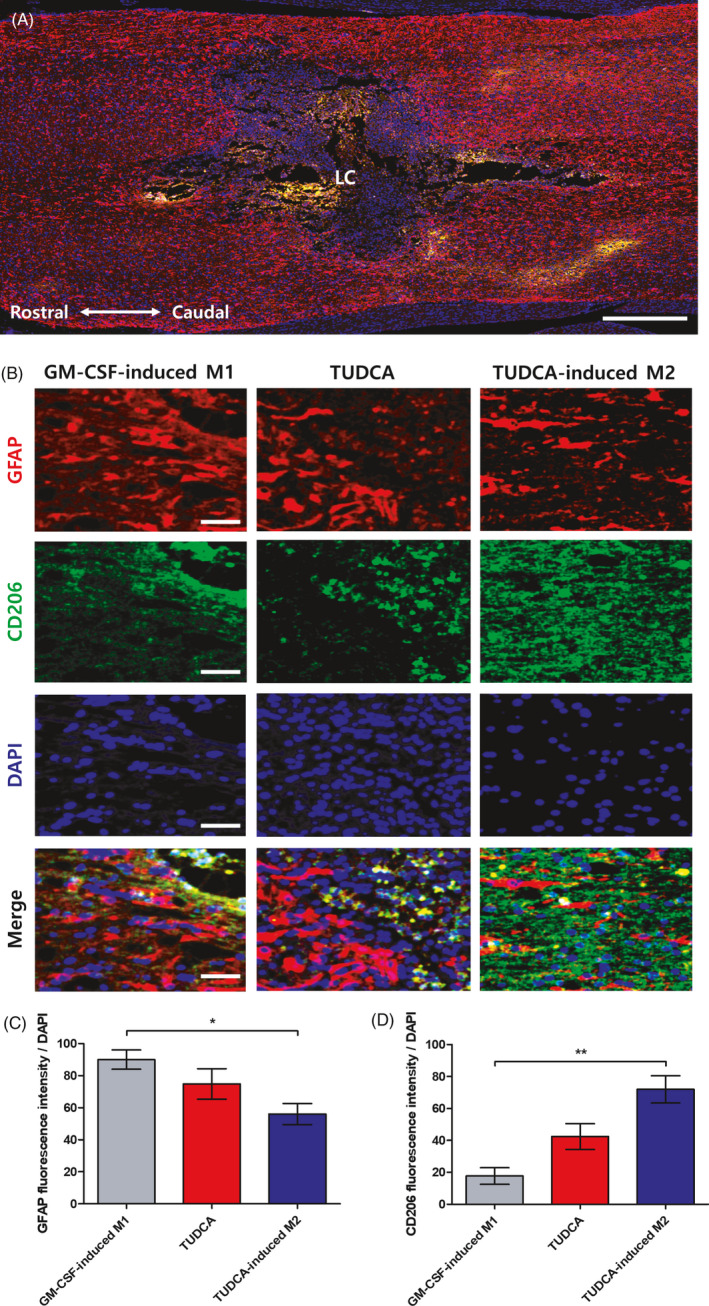
Immunofluorescence staining for glial fibrillary acidic protein (GFAP) and CD206. A, A representative image showing GFAP and CD206 of the GM‐CSF‐induced M1 group (scale bar = 500 μm). B, Expression levels of GFAP and CD206 in the GM‐CSF‐induced M1, TUDCA and TUDCA‐induced M2 groups (scale bar = 20 μm). C, Quantitative analysis of the GFAP fluorescence intensity levels. D, Quantitative analysis of the CD206 fluorescence intensity levels. Results are the mean ± SEM of triplicate experiments: ^*^P < .05 and ^**^P < .01. LC: lesion core

We evaluated whether TUDCA‐induced M2 transplantation could improve the motor function. The motor function according to the BBB hindlimb locomotor ratings was evaluated at 1, 3, 5, 7, 9, 12, 14, 16, 18 and 21 days (Figure 5C). One day after SCI, no differences were found in the extent of motor functional impairment in all groups. However, on day 16, motor function in the TUDCA‐induced M2 group was improved compared to that in the TUDCA group (Figure 5C; TUDCA group vs TUDCA‐induced M2 group: 8.5 ± 0.71 vs 10.5 ± 0.7; *^#^P* < .05). This tendency was also found at 18 days (Figure 6C; TUDCA group vs TUDCA‐induced M2 group: 7.6 ± 0.35 vs 10.5 ± 0.71; *^#^P* < .05).

### 3.5 Transplantation of TUDCA‐induced M2 macrophages inhibits GFAP and increases the expression of CD206

To evaluate the anti‐neuroinflammatory effect of the TUDCA‐induced M2 macrophages, we conducted an immunofluorescence assessment using GFAP (M1) and CD206 (M2) markers (Figure 6A,B and Figure S2A,B). Figure 6B presents the expression levels of CD206 and GFAP for each group. The GFAP/DAPI fluorescence intensity in the TUDCA‐induced M2 group was significantly decreased relative to that in the GM‐CSF‐induced M1 group (Figure 6C; GM‐CSF‐induced M1 group: 0.71 ± 0.08 and TUDCA‐induced M2 group: 0.33 ± 0.01; ^***^P < .001). The GFAP/DAPI fluorescence intensities in the sham and simple injury groups were detected at 130.60 ± 40.34 and 266.54 ± 82.32, respectively (Figure S2C). On the other hand, the CD206/DAPI fluorescence intensity in the TUDCA‐induced M2 group was significantly increased compared to that in the GM‐CSF‐induced M1 group (Figure 6D; GM‐CSF‐induced M1 group: 0.88 ± 0.19 and TUDCA‐induced M2 group: 0.29 ± 0.05; ^***^P < .001). The CD206/DAPI fluorescence intensities in the sham and simple injury groups were detected at 130.60 ± 40.34 and 266.54 ± 82.32, respectively (Figure S2D).

### 3.6 Transplantation of TUDCA‐induced M2 macrophages increases CST axons past the lesion

**FIGURE 7:**
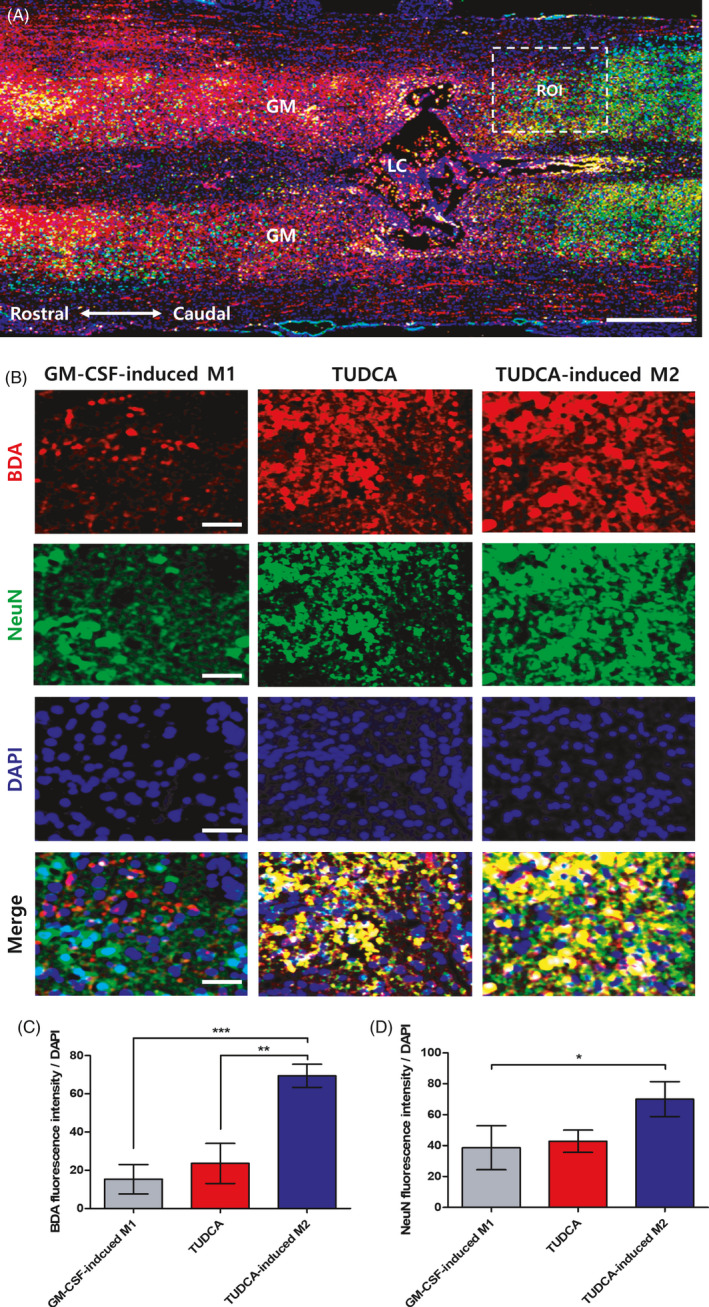
Biotinylated dextran amine (BDA) tract tracing of the corticospinal tracts and neuronal nuclear protein (NeuN) staining. A, A representative image showing BDA and NeuN of the GM‐CSF‐induced M1 group (scale bar = 200 μm). B, Expression levels of BDA and NeuN in the GM‐CSF‐induced M1, TUDCA and TUDCA‐induced M2 groups (scale bar = 20 μm). C, Quantitative analysis of the BDA fluorescence intensity levels. D, Quantitative analysis of the NeuN fluorescence intensity levels. Results are the mean ± SEM of triplicate experiments: ^*^P < .05, ^**^P < .01 and ^***^P < .001. GM: grey matter, LC: lesion core and ROI: region of interest

CST axons were traced by injecting BDA into the grey matter 7 days post‐SCI. We undertook staining using an axonal tract marker and a neuronal marker (NeuN) (Figure 7A,B and Figure S3A,B). BDA is routinely used to trace axonal tracts. NeuN has not been detected in tissues other than nervous tissues. Therefore, NeuN was considered as a specific neuronal marker.^28^ The BDA fluorescence intensity in the TUDCA‐induced M2 group was significantly increased relative to those in the GM‐CSF‐induced M1 group and the TUDCA group (Figure 7C; GM‐CSF‐induced M1 group: 15.31 ± 7.68, TUDCA group: 23.60 ± 10.47 and TUDCA‐induced M2 group: 69.46 ± 6.05; ^**^P < .01 and ^***^P < .001). The BDA fluorescence intensities in the sham and simple injury groups were detected at 69.46 ± 6.05 and 15.31 ± 7.68, respectively (Figure S3C). Moreover, the NeuN fluorescence intensity in the TUDCA‐induced M2 group was significantly increased compared to that in the GM‐CSF‐induced M1 group (Figure 7D; GM‐CSF‐induced M1 group: 38.60 ± 14.24 and TUDCA‐induced M2 group: 70.07 ± 11.31; ^*^P < .05). The NeuN fluorescence intensities in the sham and simple injury groups were detected at 109.62 ± 8.46 and 38.60 ± 11.63, respectively (Figure S3D). Specifically, there was a greater increase in the number of CST axons in the TUDCA‐induced M2 group than in the GM‐CSF‐induced M1 group and the TUDCA group.

## 4 DISCUSSION

In this study, TUDCA was found to upregulate M2 on BMDMs (Figure 2). After SCI, the TUDCA‐induced M2 macrophages decreased pro‐inflammatory cytokines (IL‐1β, TNF‐α and IL‐6) and increased the anti‐inflammatory cytokine (IL‐4) (Figure 3). Therefore, the TUDCA‐induced M2 macrophages inhibited the phosphorylation of the ERK, JNK and p38 in the MAPK signal pathway (Figure 4). The TUDCA‐induced M2 macrophages improved histopathological damage and promoted functional recovery to spinal cords in SCI rats (Figure 5). Moreover, the TUDCA‐induced M2 macrophages decreased GFAP and increased CD206 levels (Figure 6). In addition, BDA fluorescence intensity was detected at significant levels in the caudal site of the TUDCA‐induced M2 group (Figure 7).

Macrophages are key players in immunity. Macrophages express two differential phenotypes, specifically classically activated inflammatory M1 and alternatively activated anti‐inflammatory M2.^29^ After the BMDMs underwent culturing for 2 days, the M2 marker (CD206 and Arg‐1) levels in the TUDCA‐treated cells were increased to a greater extent than those in GM‐CSF‐treated cells.

M1 macrophages release pro‐inflammatory cytokines in an injured spinal cord. The pro‐inflammatory cytokines TNF‐α, IL‐1β and IL‐6 aggravate inflammation.^12^, ^30^, ^31^ M2 macrophages can affect anti‐inflammatory cytokine production in SCI.^6^, ^23^, ^32^ The transplants of TUDCA‐induced M2 macrophages showed the greatest anti‐neuroinflammatory effect in the injured spinal cord here.

The MAPK pathways of ERK, JNK and p38 are the main signal pathways in inflammation.^33^ On the injured spinal cord, the inhibition of all three MAPK pathways alleviates neuropathic pain and inflammation.^34^, ^35^ The ERK, JNK and p38 pathways induce inflammatory responses by pro‐inflammatory cytokines, that is, TNF‐α, IL‐1β and IL‐6.^36^, ^37^, ^38^ In our study, the ERK, JNK and p38 pathways in the TUDCA‐induced M2 group were more inhibited than those in the GM‐CSF‐induced M1 and TUDCA groups. In other words, the transplantation of TUDCA‐induced M2 macrophages inhibited inflammatory responses by decreasing the ERK, JNK and p38 in the MAPK pathways.

M2 macrophages have been observed in injured spinal cord and repair models.^39^ Tissue repair and regeneration are important for the survival of all living organisms.^40^ M2 macrophages release an anti‐inflammatory cytokine (IL‐4). IL‐4 is a key factor in injury repair and tissue development.^40^, ^41^ Therefore, the transplantation of TUDCA‐induced M2 macrophages into the injured spinal cord improved the tissue volume and upregulated the motor function.

SCI activates the expression of astrocytes. Activated astrocytes increase the production of GFAP. GFAP produces numerous pro‐inflammatory cytokines.^42^, ^43^ In this study, TUDCA‐induced M2 macrophages were transplanted in the injured spinal cord. The TUDCA‐induced M2 macrophages were demonstrated to be able to inhibit astrocytes and enhance the anti‐inflammatory effect.

The transplantation of TUDCA‐induced M2 macrophages shows an anti‐neuroinflammatory effect and axon regeneration in the injured spinal cord. Therefore, we suggest that the transplantation of TUDCA‐induced M2 macrophages can be a useful cell therapy after SCI.

## CONFLICT OF INTEREST

The authors declare no competing financial interest.

## AUTHOR CONTRIBUTIONS

GHH, SJK and SIS conceived the idea and conceptualized the study. GHH, SJK, WKK and DYL conducted the bioinformatics analysis and interpreted results. IBH, SHS and JBH generated the figures. GHH and SJK wrote the paper. SIS supervised the whole study process and revised the manuscript. All authors have read and approved the version of the final manuscript.

## Supporting information

Fig S1‐S3

## Data Availability

Research data are all within the present manuscript and the additional files.
