# Supplementary material for: Transplantation of tauroursodeoxycholic acid–inducing M2‐phenotype macrophages promotes an anti‐neuroinflammatory effect and functional recovery after spinal cord injury in rats
Source: Cell Prolif. 2021 May 7;54(6):e13050. doi: 10.1111/cpr.13050 (PMC8168422; doi:10.1111/cpr.13050)
Supplement: Supplementary file 1 — Fig S1‐S3 [file CPR-54-e13050-s001.docx]

**Supplementary Information for**

**Transplantation of Tauroursodeoxycholic Acid-inducing M2-phenotype Macrophages Promote an Anti-neuroinflammatory Effect and Functional Recovery after Spinal Cord Injury in Rats**

Running Head: Transplantation of TUDCA-induced M2 macrophages

Gong Ho Han ^1, 2†^, Seong Jun Kim ^1, 2†^, Wan-Kyu Ko ^1, 2^, Daye Lee ^1, 2^, In-Bo Han ^1^, Seung Hun Sheen ^1^, Je Beom Hong ^3^, Seil Sohn ^1*^

^1^ Department of Neurosurgery, CHA Bundang Medical Center, CHA University, 59, Yatap-ro, Bundang-gu, Seongnam-si, Gyeonggi-do, 13496, Republic of Korea

^2^ Department of Biomedical Science, CHA University, Seongnam-si, Gyeonggi-do, Republic of Kore

^3^ Department of Neurosurgery, Kangbuk Samsung Hospital, Sungkyunkwan University School of Medicine, Seoul, Korea

***Corresponding author:**

Seil Sohn, MD, PhD

Department of Neurosurgery,

CHA University College of Medicine

59, Yatap-ro, Bundang-gu, Seongnam-si, Gyeonggi-do, 13496, Korea

Phone: 82-31-881-7966

Fax: 82-2-780-5269

E-mail: sisohn@cha.ac.kr

^†^ Gong Ho Han and Seong Jun Kim contributed equally as the first authors


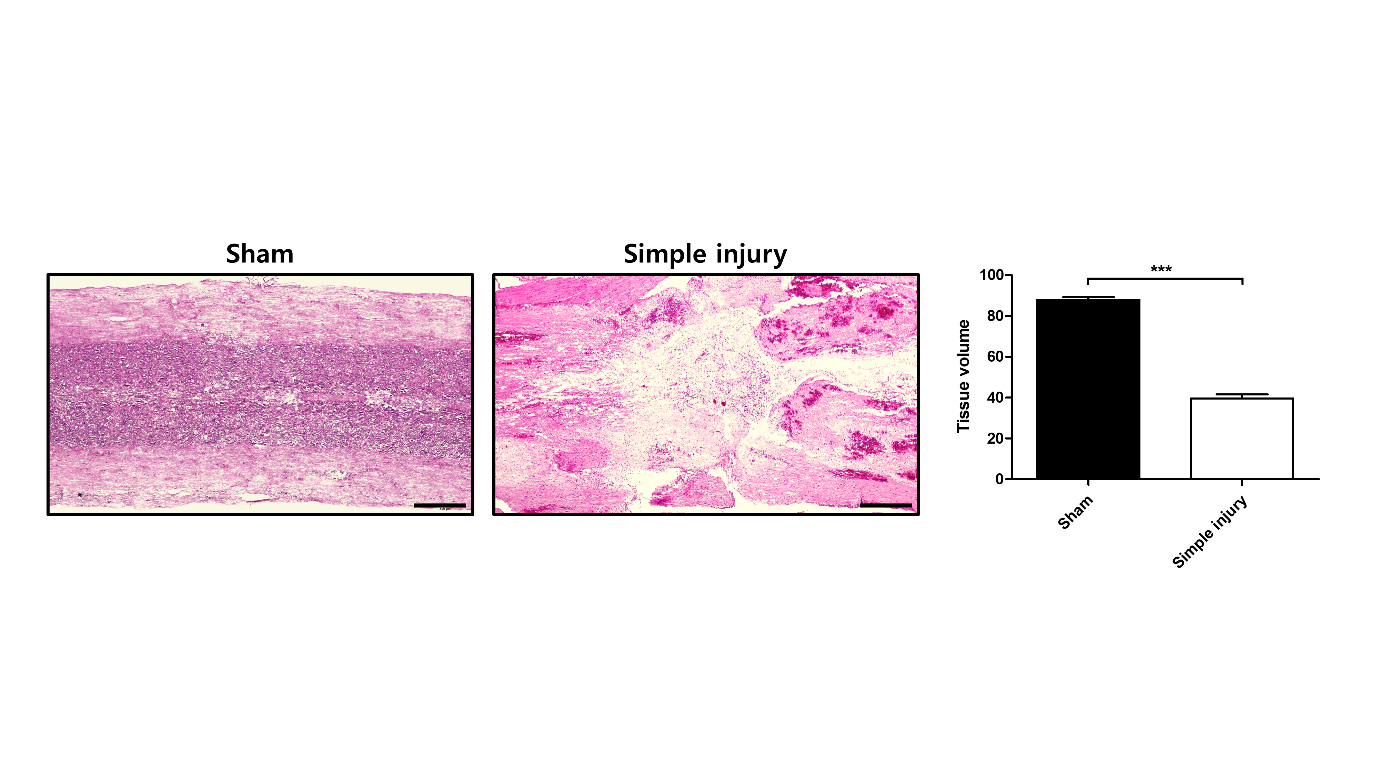


**FIGURE S1** Hematoxylin and Eosin staining. The histological structures of the tissue volume are shown in sham and simple injury groups (scale bar = 500 μm). Quantitative analysis of the tissue volume is tissue volume of the mean ± SEM of triplicate experiments *^***^p < 0.001*.

**
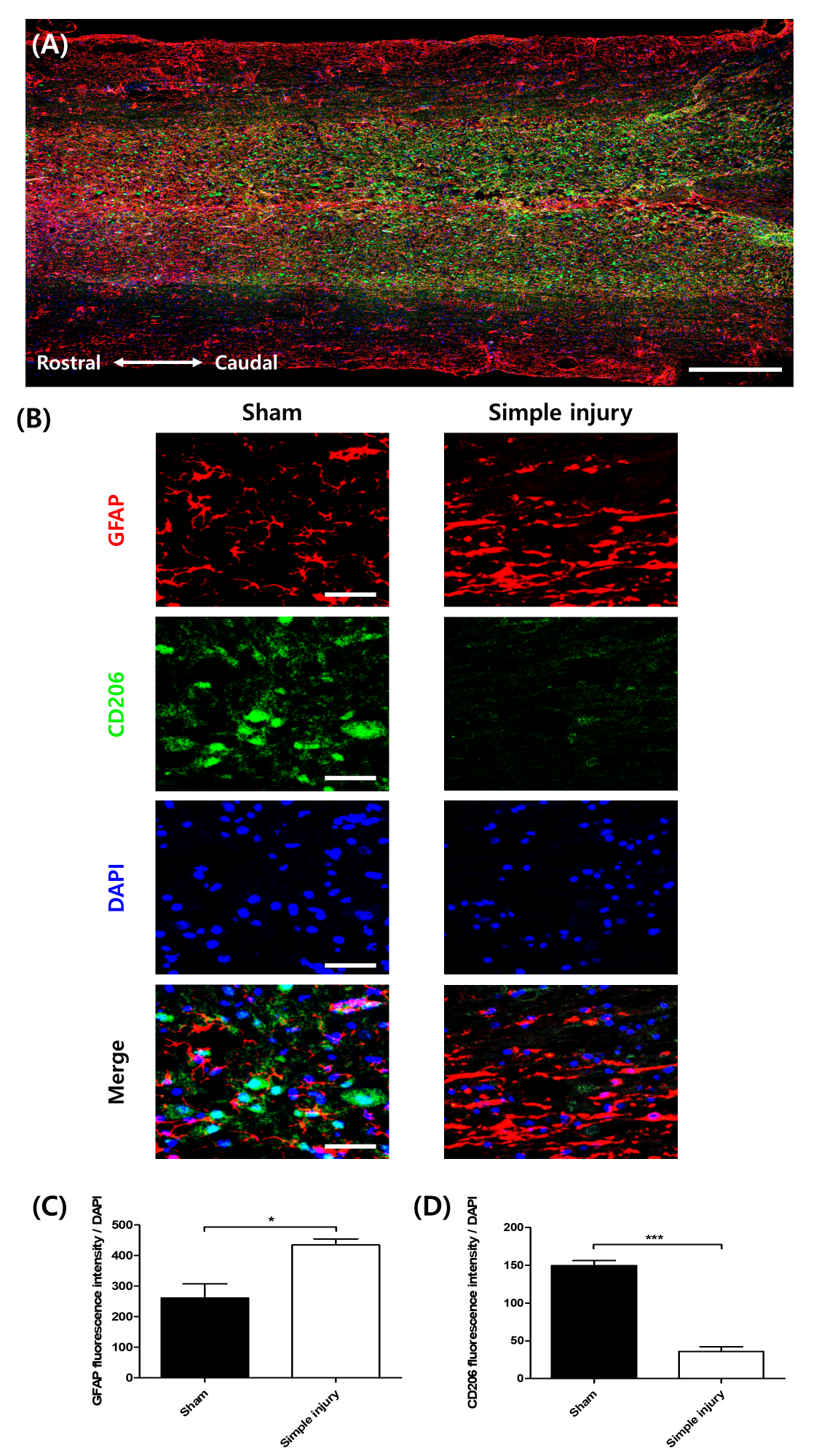
**

**FIGURE S2** Immunofluorescence staining for glial fibrillary acidic protein (GFAP) and macrophage mannose receptors (Cluster of Differentiation 206, CD206). (A) A representative image showing GFAP and CD206 of the sham group (scale bar = 500 μm). (B) Expression levels of GFAP and CD206 in the sham and simple injury groups (scale bar = 20 μm). (C) Quantitative analysis of the GFAP fluorescence intensity levels. (D) Quantitative analysis of the CD206 fluorescence intensity levels. Results are the mean ± SEM of triplicate experiments: *^*^p < 0.05* and *^***^p < 0.001*.


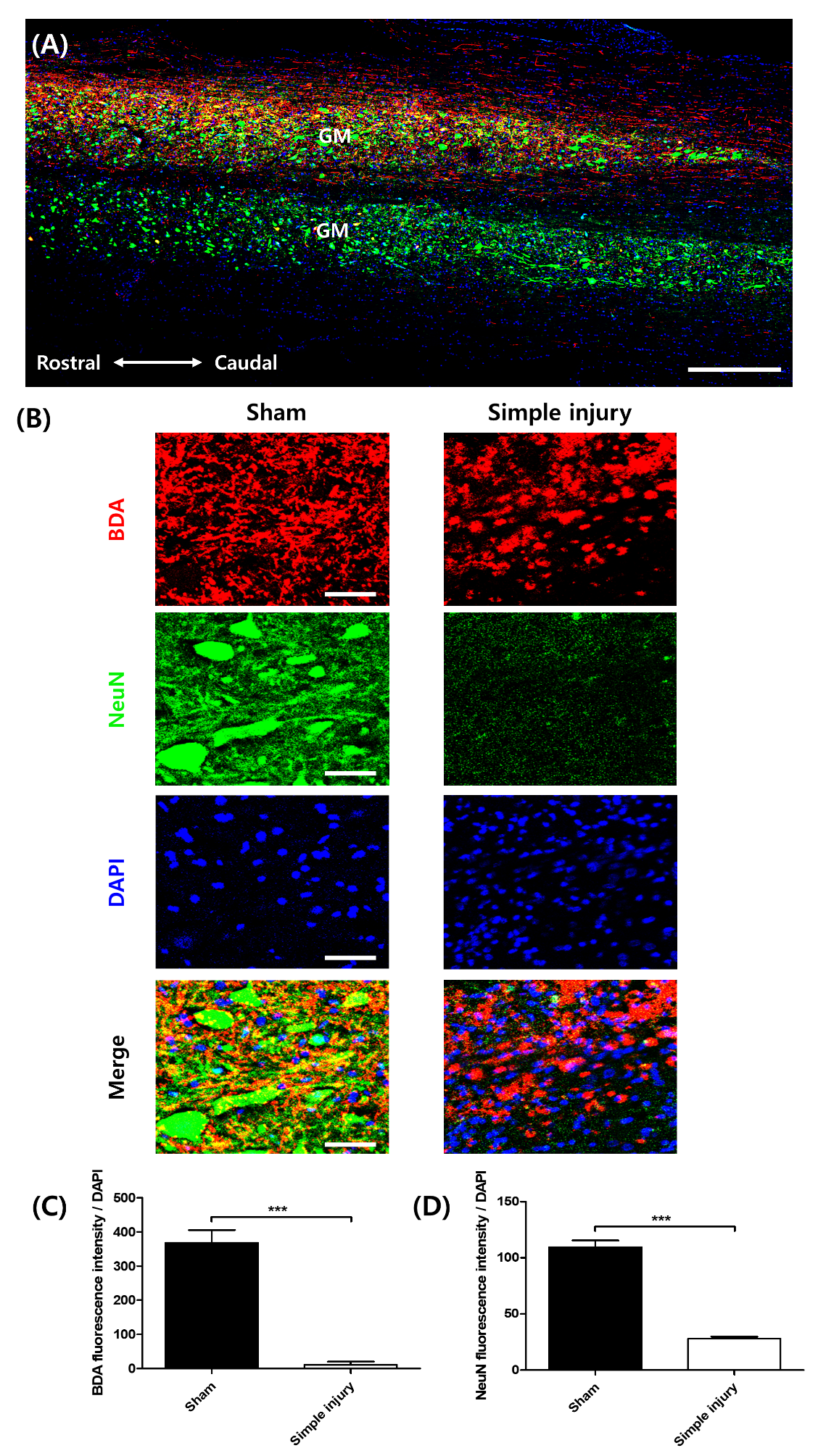


**FIGURE S3** Biotinylated dextran amines (BDA) tract-tracing of the corticospinal tracts and neuronal nuclear protein (NeuN) staining. (A) A representative image showing BDA and NeuN of the sham group (scale bar = 200 μm). (B) Expression levels of BDA and NeuN in the sham and simple injury groups (scale bar = 20 μm). (C) Quantitative analysis of the BDA fluorescence intensity levels. (D) Quantitative analysis of the NeuN fluorescence intensity levels. Results are the mean ± SEM of triplicate experiments: *^***^p < 0.001*. GM: grey matter.
